# Expansion of the Arch in the Treatment of Irregularity of the Teeth

**Published:** 1874-03

**Authors:** 


*Expansion of the Arch in the Treatment of Irregularity of the Teeth.*—Dr. S. A. Guilford, in a paper read before the Pennsylvania State Dental Society, refers to the following methods to accomplish this result:

To produce this expansion of the arch easily, rapidly and with comfort to the patient, it is only necessary to prepare a hard rubber plate closely fitting and covering the hard palate and lingual surfaces of the teeth to be moved, and inserting wooden pegs in holes drilled for the purpose in the plate just opposite the teeth. These pegs placed in the mouth dry and tightly fitting will, when wet, expand and press the teeth ; not only that, but you gain the benefit of the elasticity of the rubber plate. The advantages of such an appliance are, that it is very little in the way of the tongue ; has nothing hard in its composition to injure the teeth ; is easily removed by the patient for cleansing ; does not show from the outside, and is thoroughly effective. I have been extremely successful in the use of these plates for the past eight years.

Another means of producing the same result, where the entire superior arch needs widening, and it is desirable to do it quickly, has been proposed by Dr. Kingsley, of New York, and is, I believe, original with him. It consists of placing pieces of elastic rubber between each of the teeth and protecting the gum from the irritating effects of their impingement by placing over the roof of the mouth a thin plate of vulcanized rubber, allowing the points of the plate to extend well between the teeth at their necks. The pieces of rubber are replaced every few days by thicker pieces, until by their action the arch is so enlarged as to permit the superior teeth to overlap and properly articulate with the lower. The articulation of the teeth, aided by a thin rubber plate, accurately fitted to the roof and teeth in their new position, will permanently, it is claimed, retain the advantage gained by the wedges.

This method has the advantage of great saving of time, and if it be as free from unpleasant results as the author claims for it, it must come into general use.

